# Probing spin fluctuations of the quantum phase transition in Ce_3_Al by muon spin rotation

**DOI:** 10.1038/s41598-022-17298-6

**Published:** 2022-08-01

**Authors:** Matej Pregelj, Zurab Guguchia, Marie-Cécile de Weerd, Pascal Boulet, Stanislav Vrtnik, Janez Dolinšek

**Affiliations:** 1 J. Stefan Institute, Jamova 39, 1000 Ljubljana, Slovenia; 2 Faculty of Mathematics and Physics, University of Ljubljana, Jadranska 19, 1000 Ljubljana, Slovenia; 3 Laboratory for Muon Spin Spectroscopy, Paul Scherrer Institute, Forschungsstrasse 111, 5232 Villigen, Switzerland; 4 Institut Jean Lamour, UMR 7198 CNRS – Université de Lorraine, Campus Artem, 2 allée André Guinier, BP 50840, 54011 Nancy Cedex, France

**Keywords:** Magnetic properties and materials, Phase transitions and critical phenomena

## Abstract

We report on the dynamics of a magnetic-field-driven antiferromagnetic-to-paramagnetic quantum phase transition in monocrystalline Ce_3_Al via transverse-field muon spin rotation (TF-*µ*SR) experiments down to low temperature of $\sim$ 80 mK. The quantum phase transition is of a spin-flip type and takes place on the Ce–Al magnetic chains as a result of competition between the indirect exchange and the Zeeman interaction of the Ce moments with the external field, applied along the chain direction (also the direction of the antiferromagnetic axis). The Ce moments are not static at $T \to$ 0, but fluctuate in their direction due to the Heisenberg uncertainty principle. Upon applying the magnetic field sweep, the fluctuations exhibit the largest amplitude at the quantum critical point, manifested in a maximum of the muon transverse relaxation rate at the critical field. The quantum nature of fluctuations observed in the TF-*µ*SR experiments is reflected in the temperature independence of the average local magnetic field component along the external magnetic field at the muon stopping site(s) and the muon transverse relaxation rate within the investigated temperature range 1.5 K–80 mK. Quantum fluctuations are fast on the muon Larmor frequency scale, $\tau_{0} <$ 10^–10^ s.

A magnetic-field-driven quantum phase transition (QPT) has recently been reported in the Ce_3_Al intermetallic compound^1^, which exhibits both antiferromagnetic (AFM) ordering below $T_{N} =$ 2.6 K and heavy-fermion behavior^1–9^. The AFM transition takes place on the Ce–Al chains of the low-temperature $\gamma$-Ce_3_Al monoclinic (but very close to orthorhombic) structure (Fig. 1)^2^, while the Ce–Ce chains are nonmagnetic at low temperatures due to Kondo-compensation of the Ce magnetic moments. Both kinds of atomic chains propagate along the *a* crystallographic direction. In zero external magnetic field, a Néel-type collinear AFM ordering of the Ce moments on the Ce–Al chains was proposed by magnetic neutron scattering^5^, with the neighboring moments pointing oppositely along the *a* chain direction. The saturated moment per Ce ion amounts to 1.24$\mu_{B}$ (with $\mu_{B}$ denoting the Bohr magneton)^5^, which is strongly reduced relative to the Ce^3+^ free-ion value of 2.5$\mu_{B}$. This is a consequence of large magnetocrystalline anisotropy induced by crystal electric fields, which lock the magnetization into the (*a*,*b*) monoclinic plane. This plane is approximately the easy plane of magnetization.

**Figure 1:**
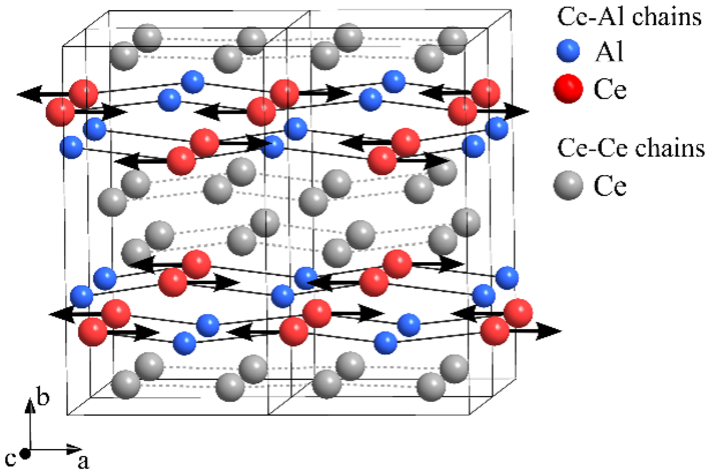
Low-temperature $\gamma$-Ce_3_Al crystal structure (adapted from ref.^2^) with the magnetic structure in zero magnetic field below 2.6 K proposed by neutron scattering^5^. A collinear antiferromagnetic ordering of Ce moments on the Ce–Al chains propagating along the *a* crystallographic direction is indicated by black arrows.

Based on the measurements of magnetic susceptibility, magnetoresistance and specific heat on a Ce_3_Al monocrystal down to $T =$ 0.35 K, it was demonstrated that the application of external magnetic field $\mathop{B}\limits^{\rightharpoonup}$ in the (*a*,*b*) easy plane drives the AFM transition continuously towards absolute zero, reaching the quantum critical point (QCP) at the critical field value of $B_{c} \approx$ 4.6 T^1^. At the QCP, a QPT from the ordered AFM state at $B < B_{c}$ to the disordered quantum paramagnetic state at $B > B_{c}$ takes place. The QPT in Ce_3_Al is considered to be of a spin-flip type^10^, where in the presence of large magnetocrystalline anisotropy, the two AFM sublattices remain polarized antiparallel until the field reaches the spin-flip field $B_{sf}$ (at $T =$ 0 equal to the critical field $B_{c}$), where the antiparallel sublattice rotates into the field direction and forms a quantum paramagnetic state (also denoted as a field-induced ferromagnetic state) for $B > B_{c}$. The magnetic-field-driven QCP in the Ce_3_Al is anisotropic with regard to the external field direction relative to the crystallographic axes. It is isotropic for the field application in the (*a*,*b*) easy plane, whereas for the field along the perpendicular *c* direction, the QCP does not occur up to 9 T (the largest applied field in ref.^1^). The reason for the QCP anisotropy is the large magnetocrystalline anisotropy.

Static (time-average) aspects of the AFM-to-paramagnetic QPT in the Ce_3_Al are detailed in the previous paper^1^. The two competing interactions that foster different ground states are the Ruderman–Kittel–Kasuya–Yosida (RKKY) indirect-exchange $${\mathcal{H}}_{ex} = - \left( {1/2} \right)\mathop \sum \limits_{i,j} {\mathcal{J}}\left( {ij} \right)\mathop{J}\limits^{\rightharpoonup} _{i} \cdot \mathop{J}\limits^{\rightharpoonup} _{j}$$ and the Zeeman interaction $${\mathcal{H}}_{Z} = - \mathop \sum \limits_{i} \mathop{\mu }\limits^{\rightharpoonup} _{i} \cdot \mathop{B}\limits^{\rightharpoonup}$$, where $$\overset{\lower0.5em\hbox{$\smash{\scriptscriptstyle\rightharpoonup}$}}{\mu } _{i} = g\mu _{B} \overset{\lower0.5em\hbox{$\smash{\scriptscriptstyle\rightharpoonup}$}}{J} _{i}$$ is the magnetic moment of the *i*th Ce ion ($$J =$$ 5/2), $$\hbar \overset{\lower0.5em\hbox{$\smash{\scriptscriptstyle\rightharpoonup}$}}{J} _{i}$$ is the angular momentum, $$g$$ is the Landé factor and $${\mathcal{J}}\left( {ij} \right) <$$ 0 is the AFM exchange coupling constant between the spins *i* and *j*. In $$B =$$ 0, the AFM state is formed below $$T_{N} =$$ 2.6 K by the exchange interaction. In a magnetic field sweep applied along the AFM axis at $$T \to$$ 0, the strength of the Zeeman interaction increases and becomes equal to the exchange interaction at the QCP ($$B = B_{c}$$), whereas for $$B > B_{c}$$, the Zeeman interaction dominates and favors the quantum paramagnetic state with the moments aligned along the field direction. Unlike the conventional thermodynamic phase transitions that occur at a nonzero temperature and are triggered by thermal fluctuations (the system does not stay in its equilibrium microscopic state, but instead randomly samples all possible states with probabilities given by the Boltzmann distribution), QPTs are triggered by quantum fluctuations since there are no thermal fluctuations left at $$T \to$$ 0. Quantum fluctuations are associated with a non-zero lowest energy (the zero-point energy) of a quantum mechanical system, because the system constantly fluctuates in its lowest energy state, as a consequence of the Heisenberg uncertainty principle^11,12^.

The QPT in an antiferromagnet described by the Hamiltonian ${\mathcal{H}} = {\mathcal{H}}_{ex} + {\mathcal{H}}_{Z}$ with Heisenberg (vector) spins^13^, including models with exchange anisotropy of an XXZ-type and quadratic single-ion crystal-field anisotropy^14,15^ has been treated theoretically for spins 1/2 and 1 in two and three dimensions on square and cubic lattices. A suppression of the Néel temperature to zero by the external magnetic field, leading to the QCP has been calculated. For the spin 5/2 pertinent to the Ce moments, such numerical calculations appear to be unfeasible due to the size of the respective Hilbert space and have not been carried out.

Spin dynamics of the QPT in the Ce_3_Al has not been studied yet. Due to the uncertainty principle, the spins cannot be static even at $T =$ 0, but undergo fluctuations around their time-average spin directions. Quantum fluctuations are of essential importance for the occurrence of the QPT. In this paper, we present a study of dynamical aspects of the quantum-fluctuations-assisted QPT in the Ce_3_Al via muon spin rotation (*µ*SR) experiments performed down to low temperature of $\sim$ 80 mK. *µ*SR is a local spectroscopic technique similar to magnetic resonances (NMR, ESR), yielding site-specific information on the static and dynamic local magnetic fields at the muon stopping sites^16^.

## Results

### Sample description and experimental setup

We have used a Ce_3_Al monocrystal, grown by the Czochralski method. The investigated sample was a 9 $\times$ 5 $\times$ 1 mm^3^ plate cut from a large crystal. It was oriented in a way that the *a* crystallographic axis (the direction of the Ce–Al magnetic chains (Fig. 1), also the direction of the AFM axis) of the low-temperature $\gamma$-Ce_3_Al monoclinic structure was perpendicular to the plate. The *µ*SR experiments were performed with surface muons that are 100% spin-polarized. The measurements were carried out in a longitudinal magnetic field (along the muon beam) applied parallel to the *a* crystal axis (i.e., perpendicular to the crystal plate), while the muons had transverse polarization. This is the appropriate geometry for the transverse-field muon spin rotation (TF-*µ*SR) experiments. Further details are given in the “Methods” section.

### TF-*µ*SR time-domain signal

**Figure 2:**
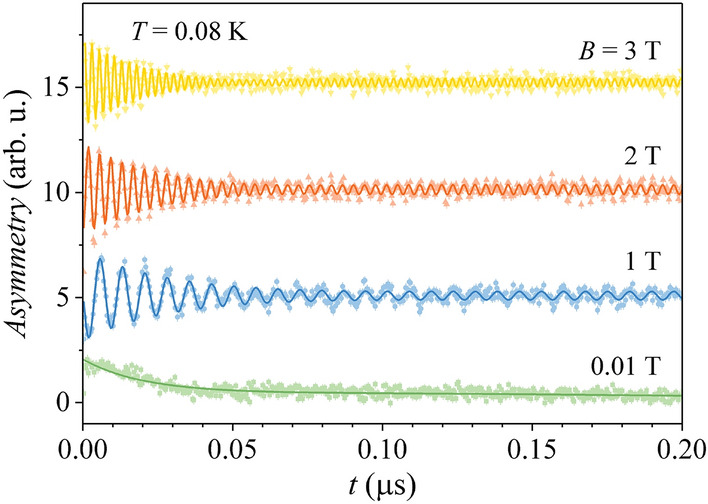
Muon spin rotation time-domain data measured at $\sim$ 80 mK in selected magnetic fields. For clarity, consecutive muon spin precession signals are offset by 5. Solid curves are fits with Eq. (1).

The magnetic-field dependence of the TF-*µ*SR time-domain signal was measured at temperatures between 1.5 K and $\sim$ 80 mK (all well below the zero-field AFM transition temperature $T_{N} =$ 2.6 K) in magnetic fields up to 8 T. The field steps were larger at low and high magnetic fields, while they were smaller near the critical field (between 4 and 5 T). The most detailed measurement was the one conducted at the lowest temperature of $\sim$ 80 mK. Representative muon spin rotation data at $T \cong$ 80 mK in selected magnetic fields are shown in Fig. 2.

**Figure 3:**
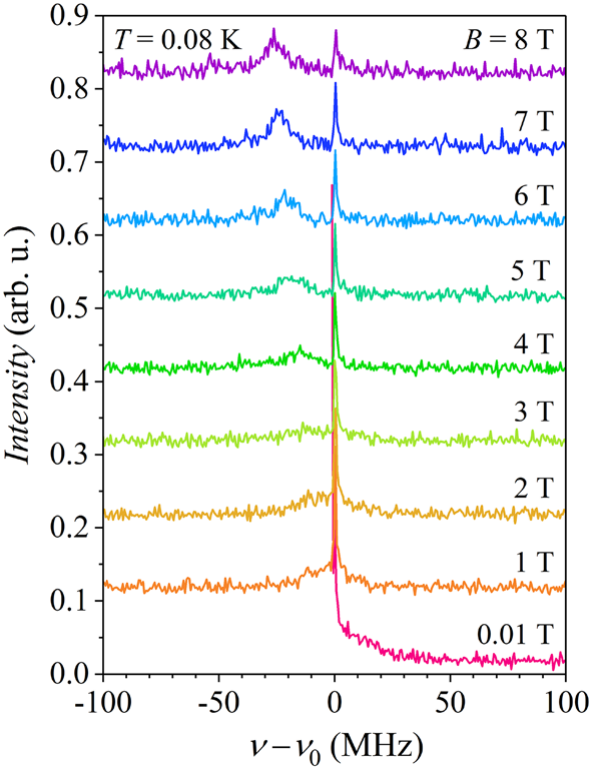
Fourier transforms of the muon spin rotation time-domain data measured at $\sim$ 80 mK in magnetic fields up to 8 T. For clarity, consecutive spectra are offset by 0.1.

The signals are composed of two contributions. The rapidly decaying signal that fully disappears in a time of $\sim$ 50 ns and accounts for about 90% of the total signal corresponds to the muons that stop in the sample, where they experience a distribution of local magnetic fields on top of the externally applied magnetic field. The remaining $\sim$ 10% of the signal with a much slower decay comes from the muons that stop in the (nonmagnetic) sample holder and rotate with the frequency almost exactly determined by the external magnetic field (the muon Larmor frequency). The local magnetic fields experienced by muons in the Ce_3_Al sample originate predominantly from the Ce–Al magnetic chains, while the nuclear magnetic fields of the order of 0.1 G can be considered negligible. Dual nature of the *µ*SR signal clearly shows up in the frequency domain (Fig. 3), where Fourier transforms of the time-domain signals from Fig. 2 are presented. For each magnetic field, the zero of the frequency scale was set at the muon Larmor frequency $\nu_{0} = \gamma_{\mu } B/2\pi$ (where $\gamma_{\mu }$ is the muon gyromagnetic ratio). The sharp peak at $\nu - \nu_{0} =$ 0 corresponds to the signal from the sample holder, while the broad peak originates from the Ce_3_Al sample.

The time-domain signals were fitted with the expression.

1 $$A = A_{1} exp\left( { - \lambda_{1} t} \right)cos\omega_{1} t + A_{sh} exp\left( { - \lambda_{sh} t} \right)cos\omega_{sh} t + A_{0} .$$

**Figure 4:**
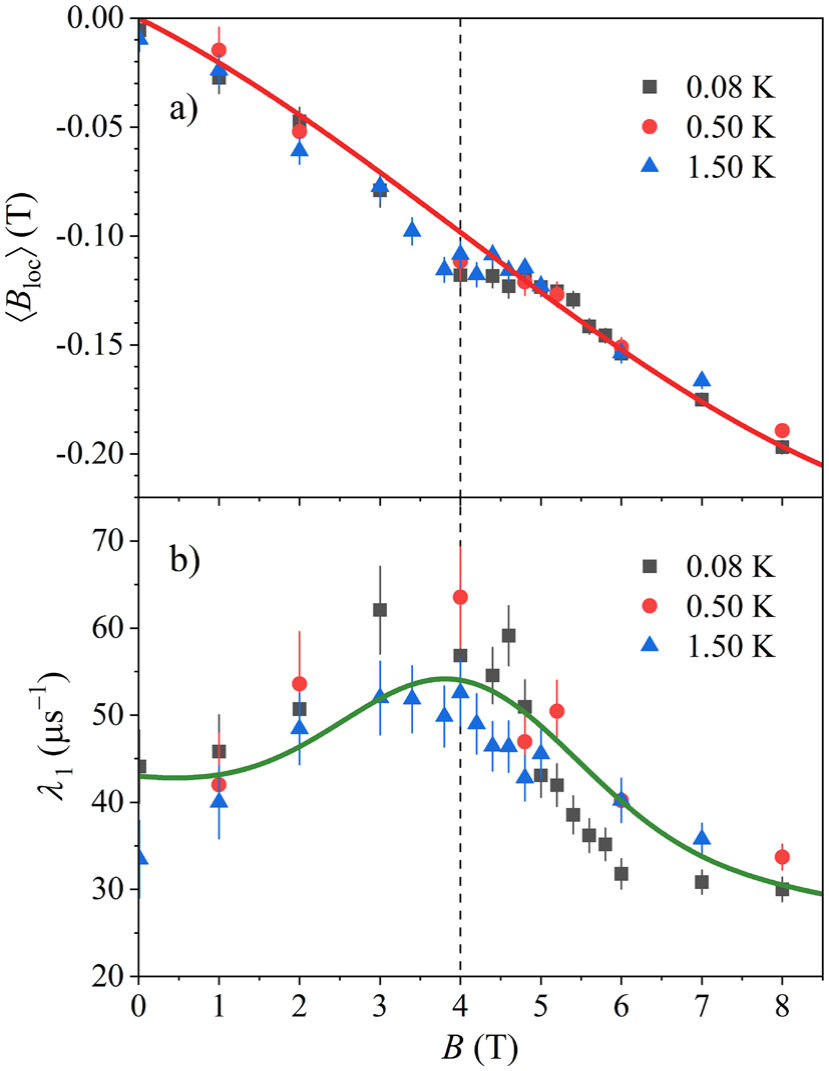
(**a**) Average local magnetic field $B_{loc} = B_{1} - B$ at the muon stopping site(s) as a function of the external magnetic field $B$. Solid curve is the fit with Eq. (2). (**b**) Muon transverse relaxation rate $\lambda_{1}$ as a function of $B$. Solid curve is the fit with Eq. (5). The dashed vertical line marks the fit-determined critical field $B_{c} =$ 4.0 T.

Here $A_{1}$ is the asymmetry of the signal coming from the Ce_3_Al sample, $A_{sh}$ is the asymmetry of the signal from the sample holder and $A_{0}$ is a (small) constant offset. The frequencies $\omega_{1}$ and $\omega_{sh}$ are the average muon spin rotation frequencies of the two signals and $\lambda_{1}$ and $\lambda_{sh}$ are the corresponding muon transverse relaxation rates. Equation (1) yielded excellent fits of the signals at all magnetic fields (solid curves in Fig. 2) by assuming that the ratio of asymmetries of the two signals, $A_{1} /A_{sh}$, does not change with the magnetic field. For the Ce_3_Al signal, we have verified that the model of single-exponential relaxation $exp\left( { - \lambda_{1} t} \right)$ yielded the best fit of the signal decays. An attempt to use stretched-exponential (i.e., multi-exponential) relaxation function $exp\left[ { - \left( {\lambda_{1} t} \right)^{\beta } } \right]$ has always yielded the stretched exponent $\beta \approx$ 1, while a Gaussian decay, as an approximation of muon spin dephasing in a static local field distribution, $exp\left( { - {\Delta }^{2} t^{2} /2} \right)$ (where ${\Delta }^{2} /\gamma_{\mu }^{2}$ is the variance of the local field distribution), didn’t give good fit of the relaxation data either. The frequency $\omega_{1}$ (converted to the magnetic field $B_{1} = \omega_{1} /\gamma_{\mu }$) and the transverse relaxation rate $\lambda_{1}$, as a function of the external magnetic field $B$ at the temperatures 1.5 K, 0.5 K and $\sim$ 80 mK are shown in Fig. 4. The following observations are evident. (1) The average local magnetic field $B_{1} - B$ at the muon stopping site(s) is independent of temperature within the investigated temperature range (Fig. 4a). At low external field $B \to$ 0, the average local field is zero, $B_{1} - B =$ 0, but then starts to grow continuously with the field (in the direction of negative fields). In the range of the QPT (roughly between 3.5 and 5 T), the $B_{1} - B$ change becomes somewhat weaker, but still noticeable. (2) The transverse relaxation rate $\lambda_{1}$ (Fig. 4b) is also temperature-independent (within the experimental precision) and shows a resonant-type character as a function of the magnetic field, by first continuously increasing at low fields, passing through a broad maximum in the region of the QPT and then decreasing upon further increase of the external field.

### TF-*µ*SR spectrum

The broad peak in the frequency-domain spectra of Fig. 3, obtained by Fourier-transforming the time-domain signals, shows the following evolution with the external field. At each field value, it is centered at the frequency $\nu_{1} = \omega_{1} /2\pi = \gamma_{\mu } B_{1} /2\pi$ determined by the average field $B_{1}$ shown in Fig. 4a. In the lowest measured external field of 0.01 T, it is centered at $\nu_{1} - \nu_{0} \approx$ 0 (hence at $B_{1} - B \approx$ 0), while at elevated fields it moves continuously to negative frequencies. The width of the broad peak also changes during the field sweep, following the field-dependent changes of $\lambda_{1}$.

## Discussion

### Magnetic-field dependence of the average local magnetic field at the muon site(s)

In the Ce_3_Al electrically conducting medium, the local magnetic field at a given muon site is a sum of three dominant terms, $B_{loc} = B_{con} + B_{trans} + B_{dip}$^17^. Here $B_{con}$ is the contact hyperfine field, originating from the Fermi contact interaction between the spins of the *s*-type conduction electrons and the muon spins; $B_{trans}$ is the transferred hyperfine field due to the RKKY indirect exchange interaction between the Ce moments via the polarization of the conduction electrons and $B_{dip}$ is the dipolar field at the muon site originating from the localized lattice (Ce) moments. $B_{trans}$ and $B_{dip}$ are sensitive to the changes in static (time-average) magnetic order and spin fluctuations on the Ce–Al chains during the external field sweep that drives the QPT.

**Figure 5:**
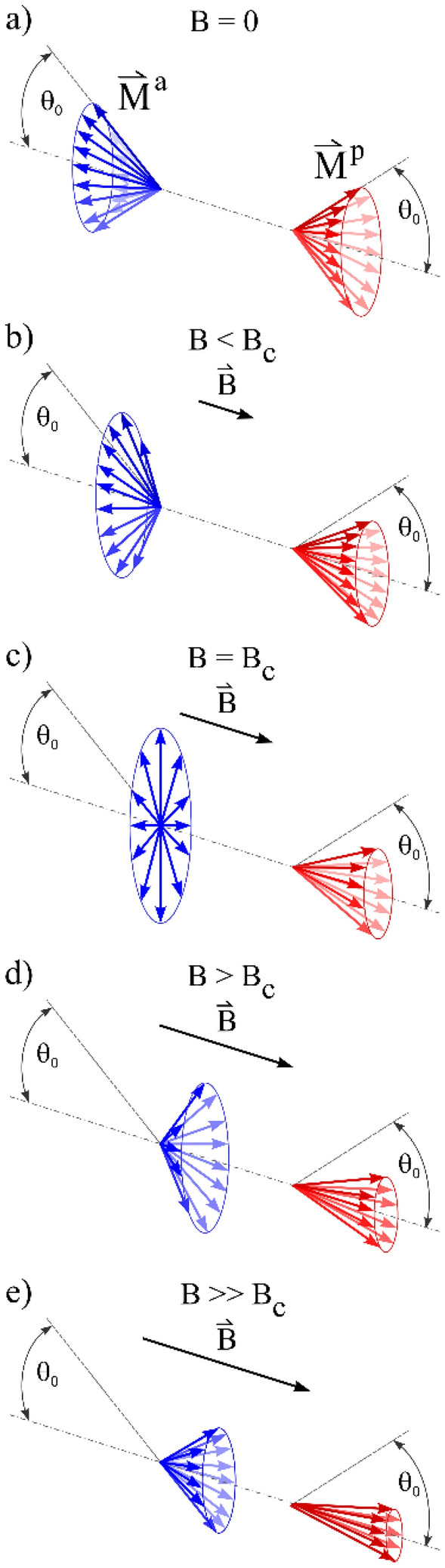
Schematic presentation of the dynamics of magnetic-field-driven AFM-to-paramagnetic quantum phase transition in Ce_3_Al for the field applied along the AFM axis. $$\overset{\lower0.5em\hbox{$\smash{\scriptscriptstyle\rightharpoonup}$}} {M} ^{p}$$ and $$\overset{\lower0.5em\hbox{$\smash{\scriptscriptstyle\rightharpoonup}$}} {M} ^{a}$$ are the sublattice magnetizations oriented parallel and antiparallel relative to the AFM axis.

The evolution of the average local field $$B_{1} - B = \left\langle {B_{{loc}} } \right\rangle$$ with the external field (the $$B_{loc} \left( B \right)$$ relation shown in Fig. 4a), where the brackets $$\left\langle \ldots \right\rangle$$ denote ensemble average over the muons, can be explained by the following qualitative dynamical picture of the magnetic-field-driven QPT. We consider that $$\overset{\lower0.5em\hbox{$\smash{\scriptscriptstyle\rightharpoonup}$}} {B} _{{loc}}$$ originates from the magnetization of the Ce–Al chains, $$\overset{\lower0.5em\hbox{$\smash{\scriptscriptstyle\rightharpoonup}$}} {B} _{{loc}} = \mu _{0} \overset{\lower0.5em\hbox{$\smash{\scriptscriptstyle\rightharpoonup}$}} {M}$$, where $$\mu_{0}$$ is the induction constant and $$\overset{\lower0.5em\hbox{$\smash{\scriptscriptstyle\rightharpoonup}$}} {M} = \overset{\lower0.5em\hbox{$\smash{\scriptscriptstyle\rightharpoonup}$}} {M} ^{a} + \overset{\lower0.5em\hbox{$\smash{\scriptscriptstyle\rightharpoonup}$}} {M} ^{p}$$ is a sum of the two oppositely polarized AFM sublattice magnetizations (of equal magnitude $$\pm M_{0}$$), treated as classical vectors. Here the superscript *p* stands for the parallel and *a* for the antiparallel magnetization direction relative to the magnetic field after the field application along the AFM axis. In zero external field at $$T \to$$ 0, the Ce moments on the two sublattices undergo time-fluctuations in the angle $$\theta$$ about their mean (time-average) directions along the Ce–Al chains, as dictated by the uncertainty principle. For simplicity we assume that the fluctuations can be treated as a stationary random process in time (an ensemble average is then equivalent to a time average) and that the spins make transitions between two values of the fluctuation angle $$\pm \theta$$ at the average fluctuation rate $$1/\tau_{0}$$ ($$\tau_{0}$$ is the average correlation time). In $$B =$$ 0, the average fluctuation angle of spins on both sublattices is equal in magnitude, $$\left\langle {\theta _{p} \left( 0 \right)} \right\rangle = - \left\langle {\theta _{a} \left( 0 \right)} \right\rangle = \theta _{0}$$. For axially symmetric fluctuations with respect to the chain direction (specified as the $$x$$ coordinate axis of a Cartesian system), the magnetizations $$\overset{\lower0.5em\hbox{$\smash{\scriptscriptstyle\rightharpoonup}$}} {M} ^{a}$$ and $$\overset{\lower0.5em\hbox{$\smash{\scriptscriptstyle\rightharpoonup}$}} {M} ^{p}$$ are located on cones making an angle $$\pm \theta_{0}$$ with the $$x$$ axis, respectively, as shown schematically in Fig. 5a. Ensemble average yields $$\left\langle {M_{{y,z}}^{a} } \right\rangle = \left\langle {M_{{y,z}}^{p} } \right\rangle = 0$$ and $$\left\langle {M_{x}^{p} } \right\rangle = - \left\langle {M_{x}^{a} } \right\rangle = M_{0} cos\theta _{0}$$, so that $$\left\langle {\overset{\lower0.5em\hbox{$\smash{\scriptscriptstyle\rightharpoonup}$}}{M} } \right\rangle = 0$$ and hence $$\left\langle {\overset{\lower0.5em\hbox{$\smash{\scriptscriptstyle\rightharpoonup}$}} {B} _{{loc}} } \right\rangle$$ 0, as appropriate for an AFM-type local field distribution. Upon the field application along $$x$$, the average fluctuation angles of spins on the parallel and antiparallel sublattices, $$\left\langle {\theta _{p} \left( B \right)} \right\rangle$$ and $$\left\langle {\theta _{a} \left( B \right)} \right\rangle$$, are no more equal. At the field values below the critical field, $$B < B_{c}$$ (Fig. 5b), spin fluctuations on the sublattice parallel to the field are progressively suppressed, $$\left\langle {\theta _{p} \left( B \right)} \right\rangle < \theta _{0}$$ (because the field tends to lock them closer to the parallel direction), while spin fluctuations on the antiparallel sublattice increase, $$\left\langle {\theta _{a} \left( B \right){\text{ }}} \right\rangle > \theta _{0}$$ (the field tends to turn over those spins from the antiparallel into the parallel direction). At the QCP ($$B = B_{c}$$), the spins on the antiparallel sublattice fluctuate equally between the parallel and antiparallel configurations (Fig. 5c), so that $$\left\langle {\theta _{a} \left( {B_{c} } \right)} \right\rangle = \pi /2$$ and consequently $$\left\langle {M_{x}^{a} \left( {B_{c} } \right)} \right\rangle = 0$$, while the average amplitude of spin fluctuations is there the largest. Above the QCP in the quantum paramagnetic phase ($$B > B_{c}$$), the spins of both sublattices point at the average into the field direction and the fluctuations progressively decrease, because the spins are increasingly stronger locked by the field. The spins of the previously antiparallel sublattice still fluctuate with a larger amplitude than those of the parallel sublattice (Fig. 5d). In the high-field limit $$B \gg B_{c}$$, both kinds of spins are strongly locked by the field, but remain to fluctuate due to the uncertainty principle (Fig. 5e).

In a simplified mathematical modeling of the experimental $\left\langle {B_{{loc}} \left( B \right)} \right\rangle$ relation shown in Fig. 4a, we consider the process depicted schematically in Fig. 5. We take the critical field value $B_{c} =$ 4.6 T from ref.^1^ as the starting value of the $B_{c}$ fit parameter and realize that the experimentally employed field range from zero to $B_{max} =$ 8 T corresponds roughly to the field range 0 $< B <$ 2 $B_{c}$. Our modeling is applicable to this field range. We further assume that the predominant change of $\left\langle {B_{{loc}} } \right\rangle$ comes from the antiparallel magnetization, $\left\langle {M_{x}^{a} \left( B \right)} \right\rangle$, which changes sign at $B_{c}$, while the field-induced change of the parallel magnetization can be, to a first approximation, neglected, $\left\langle {M_{x}^{p} \left( B \right)} \right\rangle \approx \left\langle {M_{x}^{p} \left( 0 \right)} \right\rangle$. By taking $\left\langle {\theta _{a} \left( B \right)} \right\rangle = \theta _{0} + \left( {\pi /2 - \theta _{0} } \right)\left( {B/B_{c} } \right)$ and $\left\langle {\theta _{p} \left( B \right)} \right\rangle \approx \theta _{0}$, we obtain.

2 $$\left\langle {B_{{loc}} } \right\rangle = - \mu _{0} M_{0} \left\{ { - cos\left[ {\theta _{0} + \left( {\pi /2 - \theta _{0} } \right)\left( {B/B_{c} } \right)} \right] + cos\theta _{0} } \right\}.$$

The minus sign in front of the average local magnetic field of Eq. (2) is phenomenological, following the experimental result that $B_{loc}$ is negative. Specification of the muon stopping site(s) in metallic materials is a known problem in µSR spectroscopy, which usually cannot be answered satisfactory. In the Ce_3_Al crystal, the available experimental data do not offer the possibility to specify the muon stopping site(s) in the lattice, but the experimental data are compatible with a single muon stopping site. The negative sign of the average local field at the muon site can be justified by the fact that the RKKY transferred hyperfine field $B_{trans}$ varies in space as $\left( {sin\xi - \xi cos\xi } \right)/\xi^{4}$ with $\xi = 2k_{F} r$^18^, where $k_{F}$ is the Fermi wavevector and $r$ is the distance from the Ce spin. Since $k_{F}$ is typically of the order 0.1 nm^–1^, the sign of the RKKY field fluctuates on the scale of nm and it may well be that the muon stopping site is at the position, where the RKKY field is negative. The dipolar field $B_{dip}$ also changes sign in space and can be negative at the muon position. At $B =$ 0, Eq. (2) yields vanishing average local field $B_{loc} \left( 0 \right) =$ 0. At the critical field $B_{c}$, $\theta_{a} \left( {B_{c} } \right) = \pi /2$ and $M_{x}^{a} \left( {B_{c} } \right) =$ 0, so that the average local field comes from the parallel sublattice only, $B_{loc} \left( {B_{c} } \right) \approx - \mu_{0} M_{0} cos\theta_{0}$. At $B_{max} \approx 2B_{c}$, both sublattices contribute equally to the average local field within this model, yielding $B_{loc} \left( {2B_{c} } \right) \approx - 2\mu_{0} M_{0} cos\theta_{0}$, which is twice the value at $B_{c}$. The best $B_{loc} \left( B \right)$ theoretical curve with Eq. (2), using the fit parameters $B_{c} =$ 4.0 T (quite close to the starting value from ref.^1^), $M_{0} =$ 10.5 $\times$ 10^4^ Am^–1^ and $\theta_{0} =$ 35 ± 5 deg, is shown in Fig. 4, where reasonable agreement with the experimental data is evident. Regarding the time scale of the Ce spin fluctuations on the Ce–Al chains, the above model assumes that the muon spin “sees” a time-average magnetic field $B_{1} = B + B_{loc}$ during the precession in any applied magnetic field $B$. Since $\left| {B_{loc} } \right| \ll B$, this means that the average spin fluctuation rate $1/\tau_{0}$ should be fast on the muon precession time scale. Calculating the muon Larmor frequency in the highest applied magnetic field of 8 T (using $\gamma_{\mu } =$ 851.6 Mrad s^–1^ T^–1^), we obtain $\nu_{0} = \gamma_{\mu } B/2\pi =$ 1.085 $\times$ 10^9^ s^–1^, implying that $\tau_{0} <$ 10^–10^ s. Here we neglect the fact that the correlation time may depend on the magnetic field, $\tau_{0} \left( B \right)$, being intuitively shorter at higher magnetic fields (in analogy with accelerated oscillations of a classical magnetic needle in an increasing static magnetic field), but this dependence cannot be drastic, so that $\tau_{0} \left( B \right) \approx \tau_{0} \left( 0 \right)$ appears to be a reasonable approximation.

### Magnetic-field dependence of the muon transverse relaxation rate

The field-dependence of the muon transverse relaxation rate $\lambda_{1} \left( B \right)$ from Fig. 4b can also be explained within the same picture. We adopt the spin relaxation theory by Redfield^19^ that is applicable to an ensemble of spins, which do not couple to one another, but couple to a fluctuating magnetic field, different at each spin. The assumption of uncoupled spins can be easily justified for muons (spin $I =$ 1/2), which are implanted into the sample one by one and then decay with a lifetime $\tau_{\mu } =$ 2.197 µs, so that they are unable to interact with each other. The assumption of a distribution of local fields can be justified by a spread of the muon stopping site(s), where the demagnetization effect due to the sample shape may add importantly to this distribution. The main (time-independent) muon spin Hamiltonian for the geometry of our TF-*µ*SR experiment is ${\mathcal{H}}_{0} = - \gamma_{\mu } \hslash BI_{x}$ (recall that $x$ is the direction of the external magnetic field), whereas the time-dependent perturbation that produces muon spin relaxation is ${\mathcal{H}}_{1} \left( t \right) = - \gamma_{\mu } \hslash \mathop \sum \nolimits_{q = x,y,z} B_{loc,q} \left( t \right)I_{q}$. Here $B_{loc,q} \left( t \right)$ are the components of the fluctuating local magnetic field originating from the fluctuating Ce moments that couple to the muon spin, and we again assume that $B_{loc,q} \left( t \right)$ are stationary random functions of time. We further assume that the $x$-, $y$- and $z$-components of the fluctuating field are independent. The components $B_{loc,q} \left( t \right)$ cause transverse relaxation via two effects. The distribution of the static longitudinal component $B_{loc,x}$ causes spread in the muon precession rate, while the time-dependent transverse components, $B_{loc,y}$ and $B_{loc,z}$, are effective in the relaxation when their Fourier spectrum is rich at the muon Larmor frequency $\omega_{0}$. By assuming a simple exponentially decaying correlation function of the fluctuating field with the correlation time $\tau_{0}$ (the same as defined before) for $q = x$, $y$, $z$, we get $B_{loc,q} \left( t \right)B_{loc,q} \left( {t + t^{\prime}} \right) = B_{loc,q}^{2} exp\left( { - t^{\prime}/\tau_{0} } \right)$, where $B_{loc,q}^{2}$ is the mean square fluctuation amplitude of the $q$-th component. The transverse relaxation rate is then^19^.

3 $$\lambda _{1} = \gamma _{\mu }^{2} \left[ {\left\langle {B_{{loc,x}}^{2} } \right\rangle \tau _{0} + \left\langle {B_{{loc,y,z}}^{2} } \right\rangle \tau _{0} /\left( {1 + \omega _{0}^{2} \tau _{0}^{2} } \right)} \right],$$

where $B_{loc,y,z}$ means that one of the components $B_{loc,y}$ or $B_{loc,z}$ needs to be taken (assuming that they are of equal magnitude). For a thermodynamic spin system, the fluctuation rate $\tau_{0}^{ - 1}$ depends on temperature and so does the transverse relaxation rate $\lambda_{1}$, but $\lambda_{1} \left( T \right)$ does not exhibit any maximum as a function of temperature for a monotonously increasing $\tau_{0}^{ - 1}$ upon heating (as characteristic for e.g. an Arrhenius-type thermally activated motional processes). The previously presented analysis of the $\left\langle {B_{{loc}} \left( B \right)} \right\rangle$ relation gives a strong hint that within the investigated temperature range, Ce moments fluctuate with a temperature-independent rate $\tau_{0}^{ - 1}$ in the fast-motion regime with respect to the muon Larmor frequency, $\omega_{0} \tau_{0} \ll$ 1, in which case $\lambda_{1}$ becomes.

4 $$\lambda _{1} = \gamma _{\mu }^{2} \left[ {\left\langle {B_{{loc,x}}^{2} } \right\rangle + \left\langle {B_{{loc,y,z}}^{2} } \right\rangle } \right]\tau _{0} .$$

By considering how Eq. (4) can explain the experimentally observed $\lambda_{1}$ maximum at $B \approx B_{c}$, we argue that its possible origin in the $\tau_{0} \left( B \right)$ dependence is unreasonable, because it would require an unphysical assumption of a resonant-type increase of $\tau_{0}$ (equivalent to a decrease of the fluctuation rate $1/\tau_{0}$) for the Ce moments fluctuations at the critical field of the QPT. On the other hand, the consideration that the mean square amplitude of the fluctuating transverse field component $\left\langle {B_{{loc,y,z}}^{2} } \right\rangle$ shows a resonant increase in the critical field region is realistic, because the fluctuations on the antiparallel sublattice are there the largest. We consider this effect to be the origin of the $\lambda_{1}$ maximum in the field sweep. The field-dependence of $\left\langle {B_{{loc,y,z}}^{2} } \right\rangle$ can be modeled by some bell-type function exhibiting a maximum at $B_{c}$ and we shall take it conveniently to be a Gaussian. In addition, the increased locking of fluctuations on the parallel sublattice by the increasing field (and also on the antiparallel sublattice for $B > B_{c}$) can be modeled by adding phenomenologically a linear term proportional to ${-}B$. For the fit procedure, we rewrite Eq. (4) in the form.

5 $$\lambda_{1} \left( B \right) = a_{1} - a_{2} B + a_{3} \left( {1/\sigma \sqrt {2\pi } } \right)exp\left\{ { - \left( {B - B_{c} } \right)^{2} /2\sigma^{2} } \right\}.$$

Here $a_{1}$ accounts for the muon transverse relaxation due to the spread of the static component $B_{loc,x}$, while the $a_{2}$ and $a_{3}$ terms are due to the fluctuating perpendicular component $B_{loc,y,z}$. The fit with Eq. (5) is shown in Fig. 4b by a solid curve (the values of the fit parameters are $a_{1} =$ 43 µs^–1^, $a_{2} =$ 1.6 µs^–1^ T^–1^, $a_{3} =$ 65 µs^–1^ T and $\sigma =$ 1.46 T, while we have used $B_{c} =$ 4.0 T from the fit shown in Fig. 4a), giving reasonable agreement with the experiment.

## Conclusions

To summarize, we have investigated the spin dynamics of the magnetic-field-driven AFM-to-paramagnetic QPT in the monocrystalline Ce_3_Al by the TF-*µ*SR experiments down to low temperature of $\sim$ 80 mK, where thermal fluctuations are negligible, so that the QPT is assisted by quantum fluctuations. The local magnetic fields sensed by muons originate predominantly from the Ce moments on the Ce–Al magnetic chains. The moments in an AFM configuration are not static at $T \to$ 0, but fluctuate as dictated by the uncertainty principle. The fluctuations are considered to be oscillations of the Ce moments about the AFM axis, exhibiting the largest amplitude at the quantum critical point. Quantum nature of spin fluctuations detected in the presented TF-*µ*SR experiments is reflected in the temperature independence of the observable quantities (the average local field component along the external magnetic field at the muon stopping site(s) and the muon transverse relaxation rate) within the investigated temperature range 1.5 K–80 mK. Quantum fluctuations are fast on the muon Larmor frequency scale, $\tau_{0} <$ 10^–10^ s. Regarding the possible existence of additional (“exotic”) metamagnetic states within the quantum critical region close to the QCP, the somewhat weaker $B_{loc} \left( B \right)$ dependence between about 3.5 and 5 T could indicate that “something is going on”, but in the absence of supporting theoretical calculations of magnetic structures for the Ce–Al chains with spins $J =$ 5/2, we are unable to draw conclusions beyond the speculative level.

## Methods

### Material preparation

We have grown a large Ce_3_Al crystal with the diameter of about 20 mm by the Czochralski technique, using Oxypuller 05–03 apparatus (Cyberstar S.A., Echirolles, France). The melt was prepared from a prealloyed Ce_3_Al ingot and the atmosphere in the furnace was flowing argon at ambient pressure. A Ce_3_Al seed crystal from former experiments was used and the pulling rate was 1 mm/h. The final crystal exhibited two grains along the cross section and we were able to extract a crystallographically oriented single grain that was large enough to prepare a plate of a size suitable for the *µ*SR experiments.

### Muon spin rotation experiments

The experiments were performed on the high-field *µ*SR instrument HAL-9500 ($\pi$E3 beamline) at the Paul Scherrer Institute, Switzerland, using surface muons. A BlueFors vacuum-loaded cryogen-free dilution refrigerator was used to reach the base temperature of $\sim$ 80 mK.

## Data Availability

The datasets generated and/or analysed during the current study are available in the Materials Cloud Archive repository, https://doi.org/10.5281/zenodo.6559295.
